# Visualization of gene therapy with a liver cancer-targeted adeno-associated virus 3 vector

**DOI:** 10.7150/jca.39579

**Published:** 2020-02-03

**Authors:** Xusheng Liu, Hanling Huang, Yan Gao, Lumeng Zhou, Jianwei Yang, Xiaohui Li, Yang Li, Haiwen Zhao, Shanchun Su, Changbin Ke, Zhijun Pei

**Affiliations:** 1 Department of Nuclear Medicine and Institute of Anesthesiology and Pain, Taihe Hospital, Hubei University of Medicine, Shiyan, 442000, China.; 2 Health management center, Taihe Hospital, Hubei University of Medicine, Shiyan, 442000, China.; 3 Postgraduate Training Base of Taihe Hospital, Jinzhou Medical University, Jinzhou, 121000, China.; 4 Hubei Key Laboratory of WudangLocal Chinese Medicine Research, Shiyan, 442000, China.; 5 Hubei Key Laboratory of Embryonic Stem Cell Research, Shiyan, 442000, China.

**Keywords:** Self-complementary recombinant adeno-associated virus 3, Molecular imaging, Targeted therapy, Liver cancer

## Abstract

**Background**: To evaluate the feasibility of a self-complementing recombinant adeno-associated virus 3 (scrAAV3) vector targeting liver cancer and non-invasively monitor gene therapy of liver cancer.

**Materials and methods**: An scrAAV3-HSV1-TK-kallistatin (ATK) gene drug was constructed, which contained the herpes virus thymidine kinase (HSV1-TK) reporter gene and human endogenous angiogenesis inhibitor (kallistatin) gene for non-invasive imaging of gene expression. Subcutaneous xenografted tumors of hepatoma in nude mice were generated for positron emission tomography/computed tomography (PET/CT) imaging. The ATK group was injected with the ATK gene through the tail vein, and an imaging agent was injected 2 weeks later. PET/CT imaging was performed at 1 hour after injection of the imaging agent. The control group was injected with phosphate-buffered saline at the same volume as the ATK gene drug. HE staining is used for pathological observation of tumor sections. HSV1-TK and kallistatin expression was identified by immunofluorescence, real-time quantitative PCR, and western blotting.

**Results**: Radioactivity on PET/CT images was significantly higher in the ATK group compared with the control group. 18F-FHBG uptake values of left forelegs in ATK and control groups were 0.591±0.151% and 0.017 ± 0.011% ID/g (n=5), respectively (P<0.05). After injection of the ATK gene drug, mRNA and protein expression of HSV1-TK and kallistatin in subcutaneous xenograft tumors was detected successfully. *In vitro* analysis demonstrated significant differences in the expression of HSV1-TK and kallistatin between ATK and control groups (P<0.05).

**Conclusions**: The scrAAV3 vector has a strong liver cancer-targeting ability, and the ATK gene drug can be used for targeted and non-invasive monitoring of liver cancer gene therapy.

## Introduction

Primary hepatocellular carcinoma is a common malignant tumor. There are approximately 700,000 deaths due to liver cancer each year worldwide, and about 55% occur in China, which is second only to lung cancer^1^. In recent years, immunotherapy genes, suicide gene therapy, tumor suppressor gene therapy, and antisense gene therapy for liver cancer have been reported 2,3. Gene therapy has potential for the treatment of liver cancer. Studies have shown that gene therapy with self-complementing recombinant adeno-associated virus 3 (scrAAV3) as a vector has high *in vivo* DNA stability^4^, an effective killing capacity for tumor cells 5, low toxicity and side effects^6^, and a targeting ability for liver cancer 7,8. Recombinant adeno-associated virus 3 (rAAV3) vector could selectively delivering anticancer agents to the liver cancer tissue for utilizing human hepatocyte growth factor receptor as a cellular coreceptor for binding and entry in liver cancer cells 6,9. Kallistatin is a serine protease inhibitor that has a strong inhibitory effect on angiogenesis and tumor growth 10,11. Studies have shown that overexpression of kallistatin effectively inhibits the growth of liver tumors 12,13. Traditional methods for detecting the distribution and therapeutic effects of targeted therapeutic genes in tumor tissues are often based on invasive methods. However, molecular imaging can observe the effects of gene therapy on liver cancer at an early stage and continuously *in vivo*, and it plays an important role in revealing the mechanism-of-action of targeted drugs 14,15. Therefore, it is very important to study a method for non-invasive monitoring of the dynamic distribution and curative effect of the targeted therapeutic gene *in vivo*.

Reporter gene imaging is one of the more mature molecular imaging methods 16,17. It introduces an exogenous gene (reporter gene) into cells, and the reporter gene expresses specific products such as enzymes, receptor proteins, or transporters. The technique of imaging analysis is then performed using a substrate of the radionuclide-labeled gene expression product 14,18. Molecular imaging technology of reporter genes can non-invasively, repeatedly, and quantitatively observe gene expression in living tissues, which can be used as a useful tool to detect gene transfer and distribution *in vivo*^17^,^19^, thus showing great potential for monitoring gene therapy. Herpes simplex virus type 1 thymidine kinase (HSV1-TK) is one of the most common and sensitive reporter genes 14. Furthermore, 9-[4-[^18^F] fluoro-3-(hydroxymethyl) butyl] guanine (^18^F-FHBG) is the most studied positron emission tomography (PET) imaging probe that can be combined with the HSV1-TK expression product specifically. It has a low signal, fast clearance from the blood, and good stability. ^18^F-FHBG is also a sensitive, specific, and safe gene probe that has been used in clinical research 20-22. In the previous study, we have elucidated the orchestrating role of HSV1-TK as radionuclide reporter gene imaging to monitor VEGF165 gene expression *in vivo* 23. In a very recent study, we also utilized the 18F-FHBG PET imaging with α-MHC-HSV1-tk-targeted images to monitor the differentiation of transplanted bone marrow mesenchymal stem cells successfully in myocardial infarction 24.

**Figure 1:**
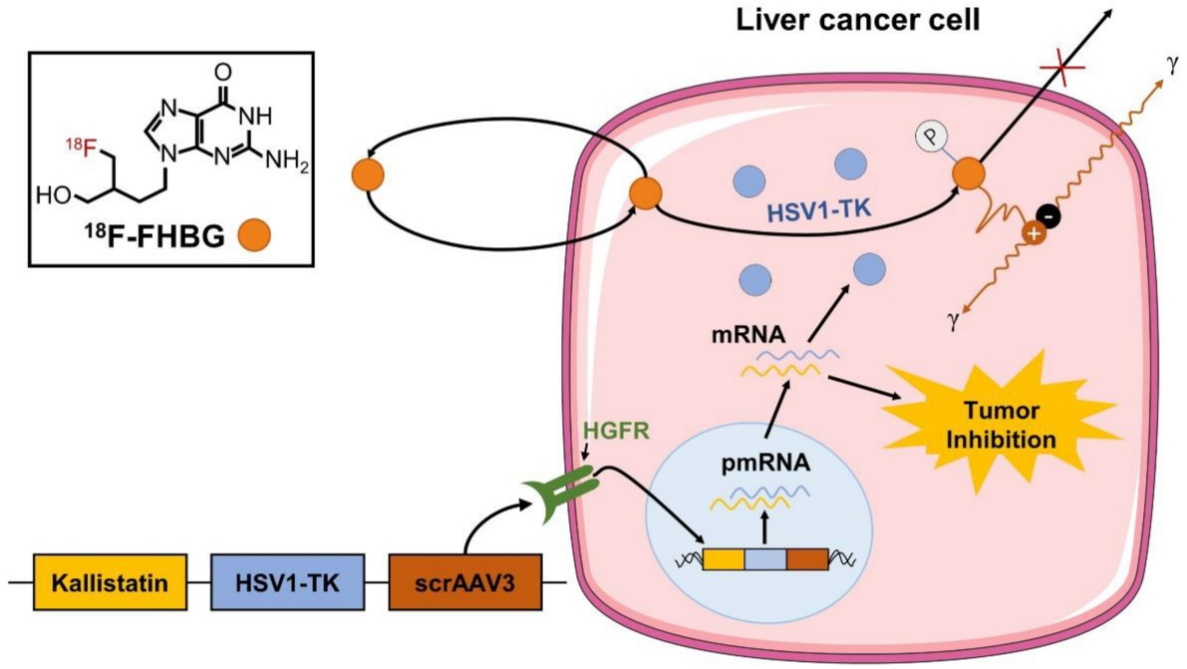
Monitoring of ATK by ^18^F-FHBG. We constructed an scrAAV3-HSV1-TK-kallistatin gene drug with a liver cancer-targeting ability. Studies have confirmed that scrAAV3 binds to HGFR and targets human hepatoma cells. The kallistatin gene inhibits neovascularization, which not only inhibits the growth of liver cancer xenografts, but also effectively inhibits metastasis and recurrence. The ATK gene drug was injected through the tail vein. Within tumor cells, HSV1-TK was transcribed and translated to produce the HSV1-TK enzyme. ^18^F-FHBG is a labeled analog of penciclovir and substrate for HSV1-TK. In the presence of HSV1-TK, the radiolabeled probe is phosphorylated and trapped within the cell. The magnitude of ^18^F-FHBG signals reflects the activity of the HSV1-TK enzyme and thus HSV1-TK gene expression.

To evaluate the feasibility of a self-complementing recombinant adeno-associated virus 3 (scrAAV3) vector targeting liver cancer and non-invasively monitor gene therapy of liver cancer, we co-constructed the HSV1-TK gene with an imaging function and therapeutic kallistatin gene in the scrAAV3 vector with a liver cancer-targeting ability to prepare the scrAAV3-HSV1-TK-kallistatin (ATK) gene drug. We were able to trap ^18^F-FHBG in liver cancer cells and image HSV1-TK reporter gene expression using ^18^F-FHBG (Fig. 1), which corresponded to ATK accumulation. For validation, we used ^18^F-FHBG/PET imaging to evaluate the distribution of therapeutic genes in tumor cells *in vivo*. Our data demonstrated that ^18^F-FHBG had high uptake in the xenograft model after ATK injection and low or no uptake in the control group. *In vitro* analytical experiments supported these results, indicating that the scrAAV3 vector may be a valuable clinical tool for targeted therapy of liver cancer.

## Materials

### Study design

The main objective of the study was to test the feasibility of visualizing an scrAAV3 vector targeting liver cancer by ^18^F-FHBG reporter gene imaging and monitoring the distribution of therapeutic genes *in vivo*. In this experiment, the ATK gene drug was designed, prepared, and injected into a subcutaneous xenograft model via the tail vein in the ATK group. The distribution of the therapeutic kallistatin gene in animal models was monitored by PET imaging, and expression of HSV1-TK and kallistatin genes was verified by immunofluorescence, real-time quantitative PCR (qPCR), and western blot analysis, thereby further confirming the liver cancer targeting of the scrAAV3 vector.

### ATK virus construction and cell culture

Gene fusion technology was used to insert HSV1-TK and kallistatin genes into the scrAAV3 vector to prepare an ATK gene drug with a liver cancer-targeting ability. Preparation and identification of the ATK gene drug was performed by VGTC (Beijing, China). HepG2 cells were purchased from the American Type Culture Collection (Manassas, VA, USA). HepG2 cells were cultured in high glucose Dulbecco's modified Eagle's medium (Hyclone, Logan, UT, USA) supplemented with 10% fetal bovine serum (Gibco), 100 U/ml penicillin, and 100 μg/ml streptomycin at 37 °C in a humidified atmosphere with 5% CO_2_.

### Animal model

All animal experimental protocols were reviewed and approved by the Animal Ethics Committee of Hubei University of Medicine (Shiyan, China). The study was carried out in accordance with the approved guidelines. Female athymic nude mice (BALB/c) at 4-6 weeks of age were housed in the specified pathogen-free experimental animal center of Hubei University of Medicine. HepG2 cells (5×10^6^) were suspended in 200 μl phosphate-buffered saline (PBS) and injected subcutaneously into the left forelegs of mice. Tumor growth was determined by electronic caliper measurements of the diameter at the inoculation site. Tumor areas were calculated by a standard formula (length × width^2^ × 0.52), where length and width are the greatest tumor diameter and greatest diameter perpendicular to the length, respectively. When the tumor area reached 100 mm^3^, 10 mice were randomly divided into ATK and control groups with 5 mice in each group. For PET imaging, the nude mice of ATK and control groups were injected with 200 μl ATK gene drug or the same volume of PBS through the tail vein, respectively. Each injection contained 1 × 10^11^ viral particles. Animals were sacrificed at 30 days after surgery.

### Preparation of ^18^F-FHBG and micro-PET/CT imaging

The ^18^F mark was prepared in accordance with a method described previously 23,24. The radiochemical purity and radiolabeled yield of ^18^F-FHBG were measured by thin layer chromatography and high performance liquid chromatography 25. Two weeks after ATK gene drug injection, mice in the ATK group were injected with 200 μCi ^18^F-FHBG through the tail vein. After 1 hour, images were acquired using a micro-PET/CT scanner (Inveon Acquisition Workplace; Siemens Medical Solutions, Germany). Animals were anesthetized with 2% isoflurane during imaging and confined to a scanning bed to prevent any movement. The PET image was reconstructed, and the same region of interest (ROI) was drawn on the left anterior leg area of the image.

### Biodistribution

Quantitative analysis of ^18^F-FHBG uptake in the intestines, brain, kidney, viscera, lung, heart, liver, spleen, muscle, and tumors was performed by PET imaging to prepare biodistribution maps.

### Hematoxylin-eosin (HE) staining and Immunofluorescence

After PET/CT imaging, the tumor tissues of nude mice were harvested and divided into four groups for immunofluorescence, HE staining, qPCR, and western blot analysis. Tumor specimens were fixed with 4% formaldehyde, embedded in paraffin and cut into 5 μm-thick sections. Hematoxylin and eosin staining (Solarbio, Beijing, China) was performed and the tissue morphology was observed under a light microscope. The *ex vivo* tumor tissue was fixed in 4% paraformaldehyde, embedded in OCT, and frozen at -20 °C. The frozen tissue was then cut into 10 μm-thick sections. The tissue sections were incubated with a mouse monoclonal antibody against TK of herpes simplex virus (1:100; GENTAUR, 1910 Kampenhout, Belgium) and rabbit polyclonal antibody against kallistatin/PI-4 (1:100; Abcam, USA) at 4°C overnight and then incubated with secondary antibodies for 30 minutes to detect protein antigens. Donkey anti-rabbit Alexa Fluor-568 and donkey anti-mouse Alexa Fluor-488 antibodies (1:400, Abcam, Cambridge, UK) were used as secondary antibodies. Nuclei were counterstained with DAPI (Beyotime, China). Immunofluorescently labeled tissues were observed under a Leica fluorescence inverted microscope.

### Real-time quantitative PCR assay

Total RNA was extracted from *ex vivo* tumor tissues using Trizol reagent (Invitrogen, Carlsbad, CA, USA). cDNA was synthesized by reverse transcription using PrimeScript™ RT Master Mix (Takara, Ohtsu, Japan). The expression levels of HSV1-TK and kallistatin genes were analyzed by qPCR using a mixture of three primer pairs (Table 1), and SYBR Green qPCR Master Mix reagent using the synthesized cDNA as a template.

### Western blot analysis

Tissues were homogenized on ice in RIPA lysis buffer (Promega, Madison, US) and centrifuged at 12,000 rpm for 15 minutes at 4 °C. The protein concentration in the supernatant was measured by a BCA assay (Beyotime, Beijing, China) at a wavelength of 595 nm. Western blot analysis was performed according to a previous study. The following antibodies were used foe western blotting: anti-HSV1-TK (1:1000), anti-kallistatin (1:1000), and anti-GAPDH (1:1000; Cell Signaling Technology). Images were obtained using a gel imaging system.

### Statistical analysis

Data are expressed as the mean ± standard deviation. Statistical analysis was performed using the SPSS 19.0 software package. Data were compared by the independent sample t-test. p < 0.05 (two-sided) was considered as statistically significant.

## Results

### Micro-PET/CT imaging

**Figure 2:**
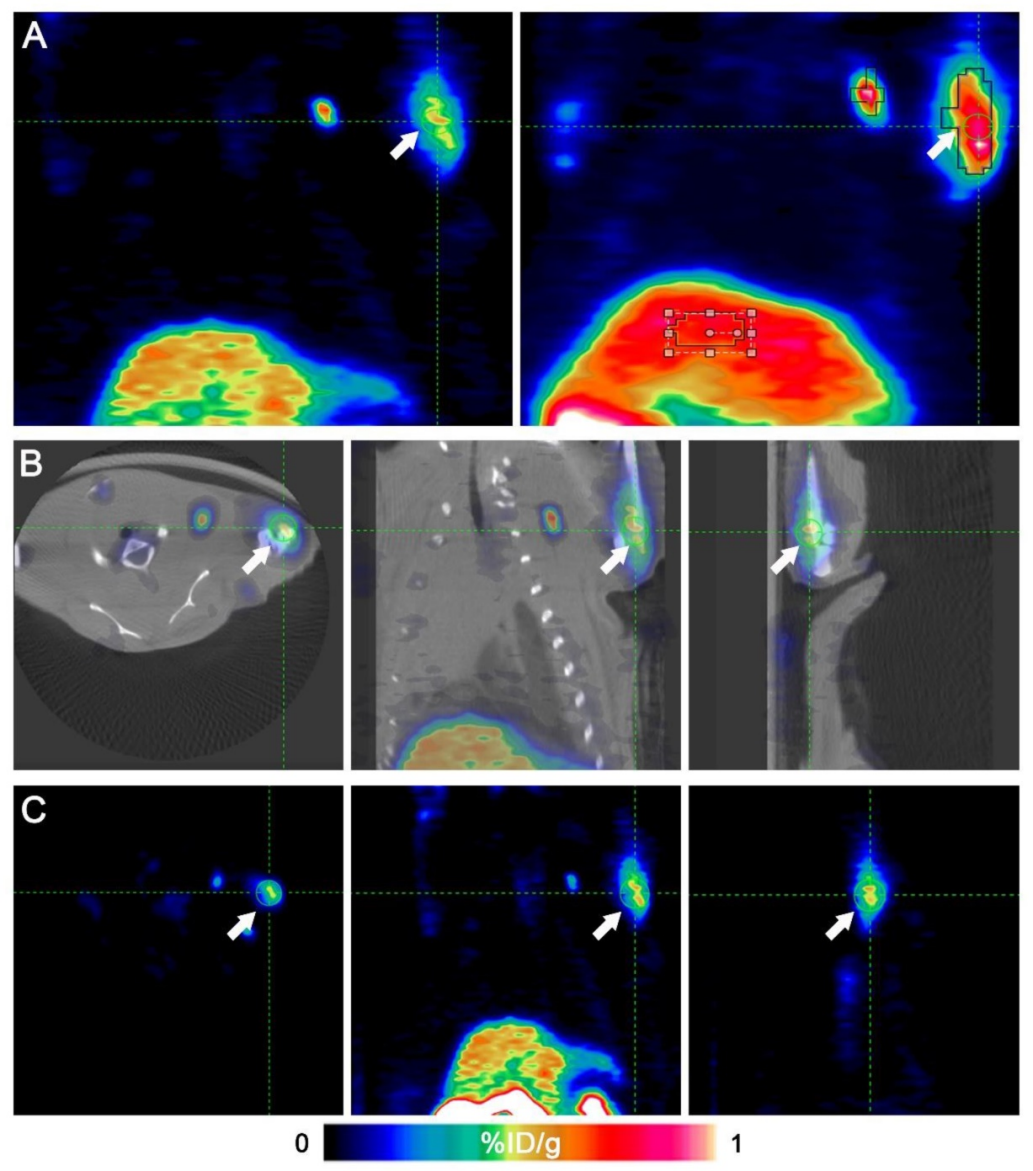
Micro-PET/CT images of a representative animal. An ^18^F-FHBG PET/CT scan was performed to detect HSV1-TK expression in the left forearm of mice. Intense HSV1-TK uptake (arrows) was observed at the left forearm. (A) Coronal slices of an animal's PET imaging (left) and image of maximum intensity projection (right). (B) Transverse, coronal and sagittal images of PET/CT. (C) Transverse, coronal and sagittal images of PET.

**Figure 3:**
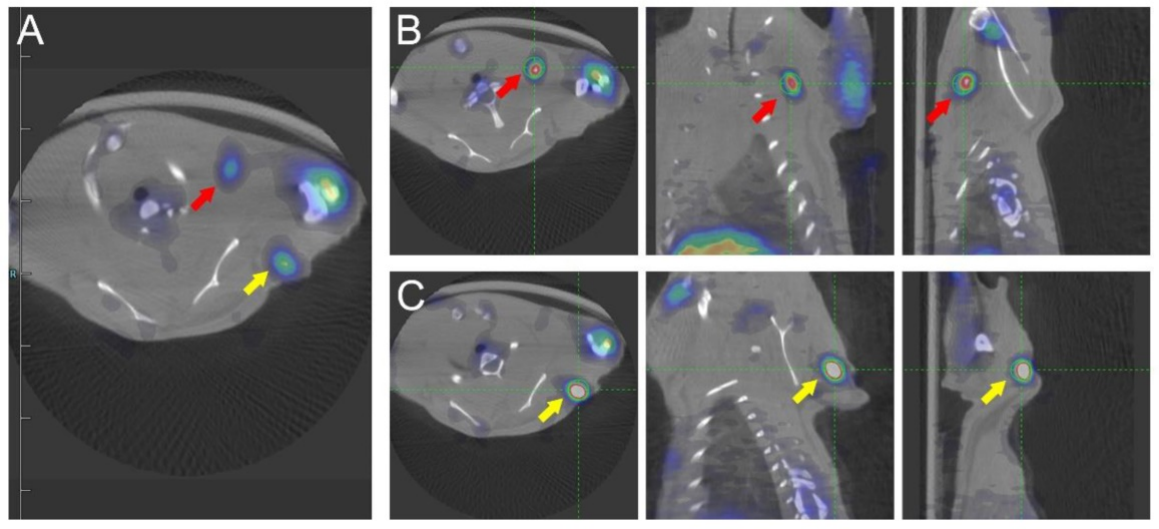
Micro-PET/CT images of transplanted metastatic lymph nodes. An ^18^F-FHBG PET/CT scan was performed to detect HSV1-TK expression in the left forearm of mice. Two additional regions of high HSV1-TK uptake were found (red and yellow arrows), indicating lymph node metastasis. (A) Transverse sections of PET/CT of metastatic lymph nodes. (B and C) Transverse, coronal, and sagittal images of PET/CT of two metastatic lymph nodes.

Fig. 2 shows that the left anterior leg injected with ATK had higher radioactivity. Identical ROIs were drawn on left forelegs of ATK and control groups, and the uptake values were 0.591 ± 0.151% and 0.017 ± 0.011% ID/g (n=5), respectively (P<0.05), indicating that the ATK gene drug carrier targeted liver cancer and can be successfully expressed *in vivo* and detected by micro-PET/CT. One of the mice in the ATK group showed significant lymph node metastasis (Fig. 3).

### Biodistribution

**Figure 4:**
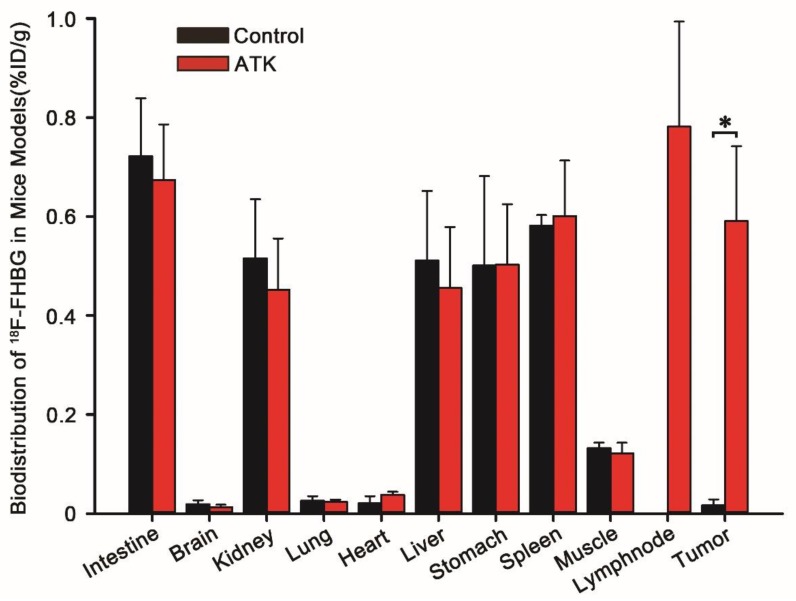
Biodistribution data of ^18^F-FHBG in xenograft-bearing mice. Semi-quantitative analysis of ^18^F-FHBG uptake by various organs. A significant difference was found in the subcutaneous xenografted tumor between experimental mice injected with the ATK gene drug and control mice without injection of the ATK gene drug (n = 5, P<0.05). However, no significant difference was observed in other organs.

The biodistribution profile of ^18^F-FHBG in mice is shown in Fig. 4. ^18^F-FHBG uptake in the intestines, brain, kidney, lung, heart, liver, stomach, spleen, and muscle of the ATK group was 0.674±0.112, 0.013±0.005, 0.452±0.104, 0.024±0.004, 0.038±0.006, 0.456±0.123, 0.503±0.122, 0.601±0.112, and 0.122±0.021 percentage of injected dose per gram (%ID/g) (n = 5), respectively, while it was 0.722±0.117, 0.019±0.008, 0.516±0.119, 0.026±0.009, 0.021±0.014, 0.511±0.141, 0.501±0.181, 0.582±0.021, and 0.132±0.011% ID/g, respectively, in the control group (n = 5). There was no difference in ^18^F-FHBG uptake between non-tumor tissues of ATK and control groups. The ^18^F-FHBG uptake value of the ATK group was significantly different from that of the control group (P < 0.05).

### *In vitro* analyses

**Figure 5:**
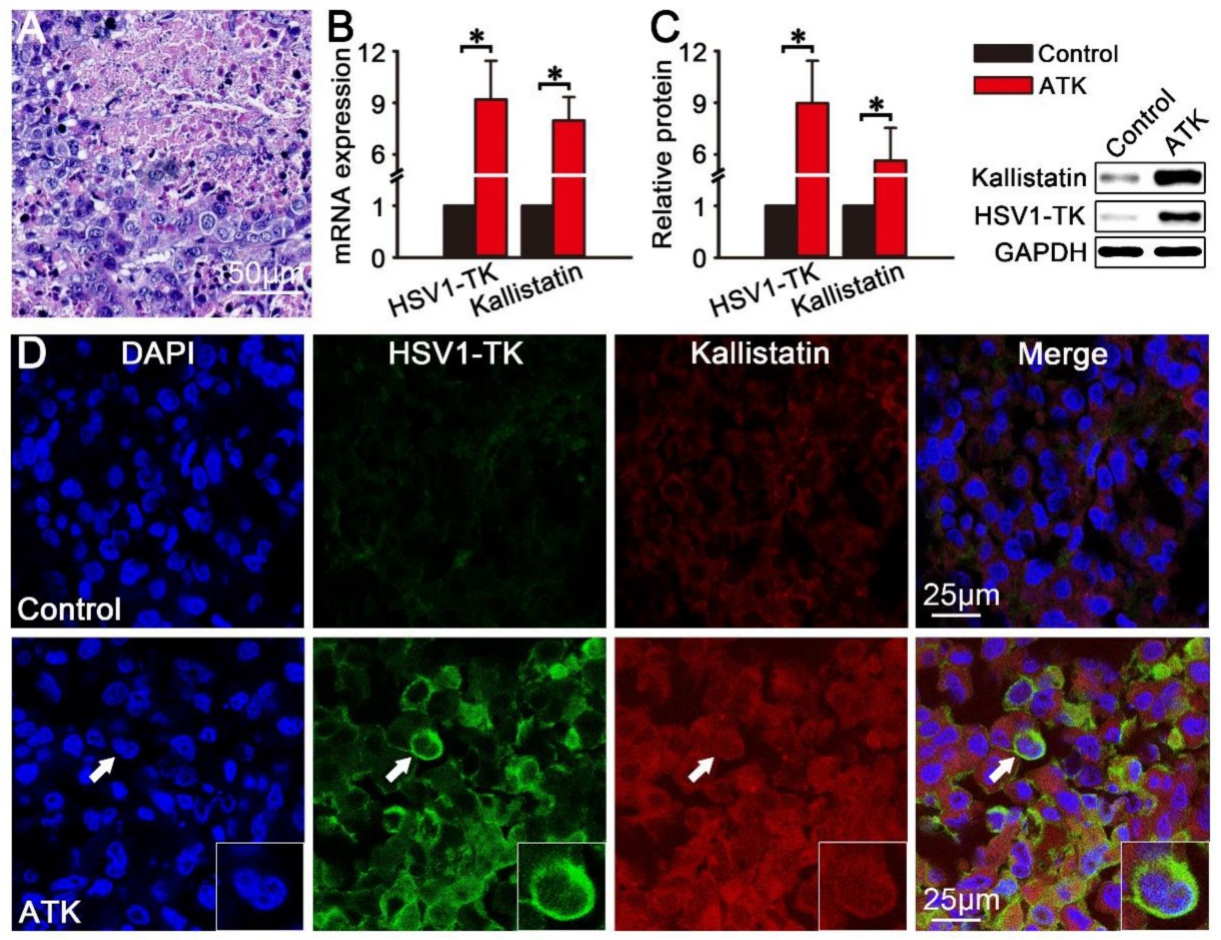
Protein and mRNA expression of HSV1-TK and kallistatin *in vitro*. The protein and mRNA expression of HSV1-TK and kallistatin in subcutaneous xenografted tumors were analyzed by immunofluorescence, qPCR, and western blotting *in vitro*. (A) HE staining results of HepG2 xenograft tumor tissues in nude mice. (B and C) Protein and mRNA expression of HSV1-TK and kallistatin of the ATK group was significantly different from that in the control group (P<0.05). (D) Immunofluorescence showed that the signals of HSV1-TK and kallistatin in the ATK group were higher than those in the control group.

After the imaging, the mice were sacrificed and tumor tissue were collected for *In vitro* analyses. After HE staining, densely packed liver cancer cells were observed under the microscope, and the results showed that the HepG2 animal model of liver cancer was successfully established (Fig. 5A). The results of qPCR (Fig. 5B) and western blotting (Fig. 5C) showed that HSV1-TK and kallistatin were significantly expressed in HepG2 xenografted tumors of the ATK group (P<0.05). Fig. 5D shows immunofluorescence images of HSV1-TK and kallistatin protein expression. The ATK group showed significant immunofluorescence of HSV1-TK and kallistatin, and the control group had lower expression.

## Discussion

It is well known that there is a close relationship between tumors and angiogenesis, and angiogenesis plays an important role in the occurrence and development of tumors 27. Upon inhibition of angiogenesis, a tumor will subside 27,28. Common angiogenesis inhibitors are angiostatin 29, platelet factor 4 30, interleukin-12 31, and matrix metalloproteinases 32. Kallistatin is a new angiogenesis inhibitor 10,11 that not only inhibits tumor growth, but also effectively inhibits their metastasis and recurrence 12,33,34. However, after administration of gene drugs, they are only relatively targeted and cannot actively recognize tumor tissues, resulting in poor gene therapy. Studies have confirmed that scrAAV3 enters cells by binding to human hepatocyte growth factor receptor (HGFR), and its scrAAV3-mediated gene drug can directly target liver cancer cells through HGFR that is highly expressed in liver cancer for good treatment results 7,8. The gene therapy of tumors can be well mediated by intratumoral injection of adenovirus. However, systemic injection of adenoviral vectors immediately accumulates in the liver and causes serious side effects 35,36. The scrAAV3 vector itself recognizes liver cancer cells and selectively delivers anticancer drugs to liver cancer with lower toxicity and side effects.

Traditionally, the therapeutic efficacy of targeted therapeutic drugs *in vivo* has been analyzed *in vitro* using invasive techniques such as *in situ* hybridization, PCR, and immunohistochemistry 7,37,38. These methods cannot dynamically monitor the expression and distribution of therapeutic genes in liver cancer cells of live animal models, and are unable to evaluate the effect of early targeted therapy or dynamically monitor at a late stage.

These deficiencies obviously do not meet the needs of *in vivo* experiments or clinical needs. Our techniques for monitoring the distribution of targeted therapeutic genes using non-invasive imaging may help overcome this limitation. Molecular imaging can visualize cellular functions and track *in vivo* molecular processes by non-invasive methods 39,40. Radionuclide reporter gene imaging is a relatively mature molecular imaging method that enables accurate and non-invasive monitoring of therapeutic genes and cells quantitatively and repetitively 14,16,17,21. ^18^F-FHBG/HSV1-TK PET imaging is currently the most common reporter gene system 14. In our previous study 41, we have performed PET/CT imaging to monitor the efficacy of gene therapy in rat breast cancer. Kim et al 42 demonstrated the safety of ^18^F-FHBG PET imaging by gene therapy and imaging of patients with recurrent gynecologic cancer. ^18^F-FHBG is a substrate for HSV1-TK that is phosphorylated by HSV1-TK and trapped within the cell 20,43. Steven et al. 44 showed the distribution of therapeutic genes in mice by luciferase optical imaging. However, these methods are restrictive in the human clinical research neighborhood. Compared with cancer specific fluorescence adenovirus-guided imaging in the examples of previous publications 44,45, ^18^F-FHBG PET reporter imaging in our study is more safety, sensitive and has been used in human clinical research 20,42.

In this study, we constructed an ATK gene drug targeting liver cancer cells. The reporter gene HSV1-TK was linked to the therapeutic gene kallistatin using a scrAAV3 vector targeting liver cancer. In our study, there was a significant difference in the uptake of ^18^F-FHBG by ATK-infected subcutaneous xenografts compared with the control group. Functional imaging and accurate anatomical positioning were obtained using ^18^F-FHBG micro-PET/CT technology. As shown in Fig. 2, we observed the precise localization of the ATK gene drug by coronal, sagittal, and cross sections. These results showed that the ATK gene drug can accurately target subcutaneous xenografted tumors of liver cancer and successfully express an active HSV1-TK enzyme. In addition, immunofluorescence results also demonstrated that both HSV1-TK and kallistatin were successfully expressed. qPCR and western blot data showed overexpression of HSV1-TK and kallistatin. We also demonstrated that the scrAAV3 vector-mediated ATK gene drug targeted liver cancer in a subcutaneous xenografted tumor of an animal model, which is consistent with previous reports. Successful expression of reporter and therapeutic genes allows continuous non-invasive monitoring of liver cancer gene therapy.

Because of the limitation of the conditions, we only performed PET imaging in small animals and did not continuously and dynamically monitor the gene therapy of the ATK gene drug targeting liver cancer. In the following study, we will perform PET imaging of ATK gene drug targeting liver cancer gene therapy in larger animals, and continuously and dynamically monitor the dynamic changes and biological distribution of ATK gene drugs *in vivo*. We will also further validate the possibility of continuous non-invasive monitoring of ATK gene drug targeting gene therapy for liver cancer using PET imaging, which may provide a powerful tool for the integration of diagnosis and treatment of liver cancer gene therapy.

## Conclusion

This is the first report of liver cancer targeted by scrAAV3 vector-mediated reporter and therapeutic genes for non-invasive monitoring of liver cancer gene therapy. Our study showed that the scrAAV3 vector has an obvious liver cancer-targeting ability, and that these optimized scrAAV3 vectors may be useful for potential targeted therapy, accurate clinical diagnosis, and *in vivo* imaging of liver cancer. Non-invasive imaging of monitoring the dynamic distribution of targeted therapeutic genes and therapeutic efficacy will aid in clinical cancer treatment.

## Figures and Tables

**Table 1:** Primer sequences for the detection of HSV1-TK and Kallistatin expression by qRT-PCR.

Gene name	Primer sequence	
HSV1-TK	Forward	5′-CTTCCGGAGGACAGACACAT-3′
	Reverse	5′-GTTTACGGGCTACTTGCCAA-3′
Kallistatin	Forward	5′-TCCTGCACACTCTCAACCTC-3′
	Reverse	5′-GAACTTCAGGTTGTGGCTCA-3′
ACTB	Forward	5′-TCTTCCAGCCTTCCTTCCT-3′
	Reverse	5′-AGCACTGTGTTGGCGTACAG-3′

## Abbreviations

scrAAV3: self-complementing recombinant adeno-associated virus 3
ATK: scrAAV3-HSV1-TK-kallistatin
PET: positron emission tomography
CT: computed tomography
^18^F-FHBG: 9-[4-[^18^F] fluoro-3-(hydroxymethyl) butyl] guanine
qPCR: real-time quantitative PCR
SD: Sprague-Dawley
PBS: phosphate-buffered saline
ROI: region of interest
HGFR: human hepatocyte growth factor receptor
pmRNA: pre-messenger RNA
